# Browning of white fat: agents and implications for beige adipose tissue to type 2 diabetes

**DOI:** 10.1007/s13105-018-0658-5

**Published:** 2018-11-30

**Authors:** A. Kaisanlahti, T. Glumoff

**Affiliations:** 1 Biocenter Oulu/Cancer Research and Translational Medicine Research Unit, University of Oulu, Aapistie 5, P.O. Box 5281, 90014 Oulu, Finland; 2 Faculty of Biochemistry and Molecular Medicine, University of Oulu, Aapistie 7A, P.O Box 5400, 90014 Oulu, Finland

**Keywords:** Brown adipose tissue, Beige adipose tissue, Type 2 diabetes, Browning

## Abstract

Mammalian adipose tissue is traditionally categorized into white and brown relating to their function and morphology: while white serves as an energy storage, brown adipose tissue acts as the heat generator maintaining the core body temperature. The most recently identified type of fat, beige adipocyte tissue, resembles brown fat by morphology and function but is developmentally more related to white. The synthesis of beige fat, so-called browning of white fat, has developed into a topical issue in diabetes and metabolism research. This is due to its favorable effect on whole-body energy metabolism and the fact that it can be recruited during adult life. Indeed, brown and beige adipose tissues have been demonstrated to play a role in glucose homeostasis, insulin sensitivity, and lipid metabolism—all factors related to pathogenesis of type 2 diabetes. Many agents capable of initiating browning have been identified so far and tested widely in humans and animal models including in vitro and in vivo experiments. Interestingly, several agents demonstrated to have browning activity are in fact secreted as adipokines from brown and beige fat tissue, suggesting a physiological relevance both in beige adipocyte recruitment processes and in maintenance of metabolic homeostasis. The newest findings on agents driving beige fat recruitment, their mechanisms, and implications on type 2 diabetes are discussed in this review.

## Introduction

Traditionally, adipose tissues have been divided into subcategories of white adipose tissue (WAT) and brown adipose tissue (BAT) according to their function and morphology. BAT functions in thermogenesis generating heat through combustion of nutrients that is uncoupled from ATP production by uncoupling protein (UCP1). Besides, the main functions of energy storage (WAT) and heat production (BAT) are considered endocrine tissues due to secretion of adipokines that participate in metabolic regulation of the body [14]. Newest addition, beige adipose tissue, has been established as an intermediate between BAT and WAT. The tissue shares the ability to thermogenesis as well as morphological features. Beige fat tissue derives from precursors of white adipocytes [27, 45] and is found in clusters scattered within the areas of white adipose tissue as opposed to occurrence of BAT in separate and distinct depots.

An aspect of interest in beige adipose tissue is that beige adipocytes have been demonstrated to be recruitable. Beige adipocytes exhibit low basal expression level of UCP1 and other thermogenesis related genes that are inducible upon stimulus, such as cold stimulation [26, 84, 91]. Theories behind the origin of beige adipocytes include (i) trans-differentiation from mature white adipocytes, (ii) existence of a distinct beige fat cell precursor, and (iii) differentiation from brown or white adipocyte precursors [17, 60, 61]. It was previously suggested that browning is a feature of WAT instead of beige adipocytes being a distinct cell type, but this theory was heavily questioned by the findings on beige adipocyte gene expression pattern by Wu et al. [88].

Brown and beige adipocytes have raised an interest in health sciences with their capability to counteract different metabolic diseases, including obesity and type 2 diabetes. Thermogenesis without ATP production, orchestrated by UCP1, is an effective way of assisting energy wastage, thus creating a negative energy balance. While some metabolic disorders and obesity are commonly linked to type 2 diabetes, brown adipose tissue contributes directly to the disease onset and permanence. Obesity resistance has also been linked to activation of these tissues in several mouse models [9, 34, 66]. In addition, brown adipose tissue might exert a beneficial effect on whole-body metabolism via other routes, such as lipid and glucose clearance from circulation. Taking into account the beneficial effects of BAT occurrence, the emergence of thermogenesis-able beige adipocytes and browning of WAT hold an interest and potential towards treating type 2 diabetes. In addition, beige adipocytes are hypothesized to have a distinct role in metabolic regulation and exhibit different physiological purposes compared to classical brown adipocytes [45]. This review discusses different browning agents that have been discovered and studied so far in the context of diabetes type 2 treatment and/or effect of browning to glucose metabolism and insulin sensitivity.

## Development of adipose tissues on the molecular level

Brown and white fat develop from mesoderm through separate differentiating lineages. Mesenchymal stem cells (MSCs; multipotent stem cells of the adipose tissue extracellular matrix) can commit to either adipogenic or myogenic lineage, leading to emergence of either white adipocytes, brown adipocytes, or myocytes. Classical brown adipocytes and myocytes develop along myogenic lineage and derive from myogenic factor 5 positive cells (*Myof5*^+^), whereas precursors of white and beige adipocytes are *Myof5*^−^ and thus adipogenic [60, 67]. Despite sharing a precursor, beige fat cells exhibit distinct gene expression compared to either WAT or BAT, combining expression pattern of genes characteristic to WAT (adiponectin, adipocyte protein 2 gene (*aP2)*, adipsin) and BAT (*Ucp1*, *Cidea*, *Pgc-1α*). In addition, beige fat cells express genes specific to the fat tissue, such as a developmental transcription factor T-box1 (*Tbx1)*, a fatty acid transporter solute carrier family 27 member 1 (*Slc27a1*), and transmembrane protein 26 (*TMEM26*) as well as molecules of immune and inflammatory response pathways, such as *CD40* and *CD137* [88]. Distinct genetic loci drive the regulation of BAT and beige adipocytes, suggesting that different pathways are responsible for regulation of these tissues.

The differentiation processes of adipogenic and myogenic lineages are orchestrated with surprisingly similar transcriptional cascades. Peroxisome proliferator-activated receptor-γ (PPARγ) is the key switch determining the cell fate and differentiation to either a brown or a white adipocyte [29]. PPARγ drives the adipogenesis transcriptional program in co-operation with a set of different co-activators and regulators, such as CCAAT/enhancer binding proteins (C/EBS), PPARγ co-activator-1α (PGC-1α) and PRDM16. PGC-1α is the major regulator of mitochondrial biogenesis and oxidative metabolism in brown fat and other cell types, such as skeletal muscle, and one of the most important factors of brown adipocyte development [62]. PRDM16, a transcriptional co-regulator, is one of the key switches between determining the development into a brown adipocyte or a myocyte. PRDM16 functions by interacting with multiple DNA-binding transcriptional factors, such as PPARα, PPARγ, and several C/EBP protein family members and has the capability of inducing PGC-1α expression, while C/EBS participate in the upkeep of the differentiation state [29, 67]. In addition to major transcriptional switches PPARγ and PRDM16 that determine the cell fate between myocyte, WAT, and BAT, other overlapping cascades and molecular factors are also involved in adipogenesis and adipocyte differentiation.

## BAT and beige connections to type 2 diabetes

BAT intakes glucose and lipids from circulation aiding in glucose clearance and relieving the demand for insulin secretion by β-cells, thus improving their function [45]. Fasting glucose concentrations in individuals with detectable BAT were demonstrated to be lower than in those without active BAT [38]. The predominant source of energy for brown adipose tissue metabolism is fatty acids stored in brown adipocytes [46], while only 10% of the energy is derived from plasma glucose. However, the clearance of this glucose has been demonstrated to be effective in diabetics. In humans, boosting of glucose uptake into BAT consists of two different mechanisms: insulin-dependent and insulin-independent [57]. Heat production-related glucose uptake into BAT has been suggested to be independent from insulin signaling, thus bypassing all the normal parameters regulating the blood glucose level [15, 50, 56]. BAT is one of the most sensitive target tissues for insulin action and is considered to be the most important metabolically active organ for glucose. BAT activation enhances insulin signaling in BAT itself by combining the insulin-independent glucose uptake with thermogenesis and uptake of the glucose administered by insulin signaling [45].

Many of the effects responsible for glucose and lipid clearance from plasma are indirectly improving the state of insulin resistance and β-cell failure. While glucolipotoxicity is one of the major pathologies behind insulin resistance, peripheral lipid and glucose clearance benefits β-cells and aids in restoring peripheral insulin sensitivity [59]. In practice, BAT-related treatment methods include (i) increasing the BAT mass, (ii) recruitment of beige adipocytes by browning of WAT, and (iii) boosting the effects of activation of already existing BAT by targeting the regulation of fuel uptake and utilization [57]. Formation of beige adipocytes has been demonstrated to have ameliorating effects to body weight as well as to glucose and lipid homeostasis [7, 50]. Physiological stimuli traditionally considered to induce the formation of beige adipocytes include cold exposure, diet, and exercise. In addition to activation of classical BAT, prolonged cold exposure, as well as β-adrenergic agonist treatment, has been demonstrated to be able to brown WAT [24, 25, 91].

## Browning agents

In the following paragraphs and Table 1, we list and discuss a comprehensive and updated set of browning agents as reported in the literature.

**Table 1:** White adipose tissue browning agents

Factor	Publication	Key findings
Cold exposure	Young et al. 1984	Cold induction leads to the emergence of *Ucp1* positive cells and morphological changes in mouse parametrial fat bad
	Cousin et al. 1992	Cold exposure increases UCP1 expression in rodent periovarian WAT
	Wang and Wahl 2014	Cold exposure stimulates BAT insulin signaling in rats
CL 316243	Ghorbani et al. 1997	Chronic treatment with CL 316243 leads to occurrence of brown adipocytes within traditional WAT depots
BRL 26830A	Cousin et al. 1992	Treatment with BRL 26830A increases UCP1 expression in periovarian WAT depots in rodents
Acetate	Sahuri-Arisoylu et al. 2016	Acetate treatment show WAT browning capacity in mice
Capsaicin	Baskaran et al. 2016	Capsaicin initiates WAT browning in mice by several different mechanisms
Resveratrol	Azhan et al. 2016	Resveratrol induces Ucp1 and Pgc1α gene expression in mice
Berberine	Zhang et al. 2014	Berberine induces development of brown-like adipocytes in inguinal WAT depot in mice
Fish oil	Kim et al. 2015	Fish oil intake lead to upregulation of UCP1 and the β3 adrenergic receptor in inguinal WAT of mice
Decaffeinated green tea extract	Chen et al. 2017	Decaffeinated green tea extract induces expression of variety of browning related biomarkers
Cinnamon	Kwan et al. 2017	Cinnamon effects UCP1 expression in adipocytes isolated from mouse subcutaneous WAT
Ginsenodise Rb1 Curcumin Quercetin	Mu et al. 2015 Lone et al. 2016 Lee et al. 2017	Ginsenoside Rb1, curcumin and quercetin promote mRNA expression of BAT markers in 3T3-L1 cells
Farnesoid X receptor	Fang et al. 2014	Farnesoid X receptor activation enhance thermogenesis and browning of subcutaneous WAT
Liver X receptors	Miao et al. 2015	Liver X receptors regulate the browning of WAT, activation of mitochondria, and increased energy expenditure
MicroRNAs	Shamsi et al. 2017	MicroRNAs regulate both BAT activation and subcutaneous WAT browning in cold exposure
	Ng et al. 2017	miRNA-32 has a role in cold-induced WAT browning
Thiazolidinediones	Petrovic et al. 2010	Chronic treatment with thiazolidinediones (TZDs) induces browning of WAT by activating PPARγ and PRDM16
Prostaglandin E2	Garcia-Alonso et al. 2014	Prostaglandin E2 diverts preadipocyte differentiation in WAT to beige adipocytes
Gleevec	Choi et al. 2016	Gleevec increases brown/beige fat thermogenic and mitochondrial genes in WAT and interscapular BAT in mice
Beta-lapachone	Choi et al. 2016	Beta-lapachone stimulates browning of WAT and increases expression of UCP1 in high-fat diet fed mice
Slit 2 derived secretory product	Svensson et al. 2016	Slit 2 derived secretory product regulates beige adipocyte induction via Prdm16 and activation of PKA signaling
Artepillin C	Nishikawa et al. 2016	Artepillin C induces brown-like adipocytes in mice and primary inguinal WAT derived adipocytes in vitro
Adrenomedullin 2	Zhang et al. 2016	Adrenomedullin 2 induces browning of rat primary adipocytes in vitro
IL-6	Abdullahi et al. 2017	Il-6 originated from bone marrow in burn injury regulates WAT browning
IL-4	Nguyen et al. 2011	IL-4 exerts a macrophage-dependent role in BAT activation during cold exposure
IEX-1	Shahid et al. 2016	Deficiency of IEX-1 lead to WAT browning via alternative activation of macrophages
Thyroid hormones	Medina-Gomez et al. 2008	Thyroid hormone metabolite TRIAC induces UCP1 expression in abdominal WAT of rats
	Lee et al. 2012	Treatment of human adipose-derived stem cells with T3 induces UCP1 expression and mitochondrial biogenesis
	Kir et al. 2014 Thomas and Mitch 2017	Parathyroid hormone (PTHr) and tumor-derived parathyroid hormone-related protein (PTHrP) administration leads to WAT browning in mice cancer-cachexia models
	Weiner et al. 2017	Thyroid hormones induce WAT browning through both peripheral and central mechanisms
Glucagon-like peptide 1	Beiroa et al. 2014 Lopez et al. 2015	GLP1 stimulates WAT browning and stimulates BAT thermogenesis in mice
Leptin	Dodd et al. 2015	Leptin drives WAT browning through action on hypothalamic neurons
Melatonin	Jiménez-Aranda et al. 2013	Melatonin induces WAT browning on inguinal WAT in rats
Natriuretic peptides	Bordicchia et al. 2012 Liu et al. 2018	Natriuretic peptides demonstrate WAT browning capacity with several different mechanisms
PTEN Cox2 Foxc2 Folliculin Gq	Klepac et al. 2016	Distinct gene set is associated with WAT browning, including PTEN, Cox2, Foxc2, Folliculin, and Gq
TGF-β/Smad3	Yaday and Rane 2012	Modulations in TGF-β/Smad3 signaling activate BAT-like phenotype in rodent WAT
FGF21	Fisher et al. 2012	FGF21 expression is essential for cold-induced recruitment of beige adipocytes
	Keipert and Jastroch 2014	FGF21 secretion from skeletal muscle leads to increased browning of epididymal and subcutaneous fat depots in mice
Apelin	Than et al. 2015	Apelin secreted from brown and white adipocytes stimulates adipose browning
Exercise	Wu et al. 1999 Villena et al. 2002	Exercise induces expression of PGC-1α
	Boström et al. 2012 Lee et al. 2014	Irisin released from muscle during exercise induces WAT browning in human and rodents
PPAR agonists	Fukui et al. 2000 Laplante et al. 2003	Treatment with PPAR agonists increases UCP1 expression in different WAT depots
	Ohno et al. 2012	Browning effect of PPAR ligands is centered to stabilization and expression of PRDM16
	Petrovic et al. 2010	Rosiglitazone treatment induces browning of epididymal WAT depot in mice
	Zhao et al. 2016	α/β-Hydrolase domain 6 negatively regulates adipose tissue browning
BMPs	Tseng et al. 2008 Shen et al. 2009	BMP7 is able to induce BAT selective genes in preadipocytes and drive browning of mesenchymal progenitor cells
	Elsen et al. 2014	BMP7 and BMP4 induce white-to-brown shifting in primary human adipose stem cells
	Ross et al. 2000	Wingless pathway (Wnt) activation represses brown and white adipogenesis by suppressing the induction of PPARγ and C/EBSα
Lactate β-hydroxybutyrate	Carrière et al. 2014	Lactate and β-hydroxybutyrate increase UCP1 gene expression in human and murine WAT
Beta-aminoisobutyric acid (BAIBA)	Roberts et al. 2014	BAIBA increases BAT marker genes in WAT of mice both in vitro and in vivo and BAT-like phenotype in human pluripotent stem cells
Retinoic acid	Wang et al. 2017	Retinoic acid induces WAT browning through activation of vascular endothelial growth factor (VEGF) signaling in vitro

### Cold exposure

Cold exposure is one of the oldest and most studied physiological stimuli leading to WAT browning. In early studies in rodents, both acute and chronic cold exposure has been reported to increase oxygen consumption, stimulate BAT insulin signaling, and to lead to the emergence of Ucp1-positive cells and morphological changes in mouse parametrial fat bad [26, 84, 91]. Cousin et al. (1992) demonstrated increase in UCP1 expression in rodent periovarian WAT in response to prolonged cold exposure in + 4 °C [13]. Cold exposure induced browning in mice has also been studied by Vitali et al. (2012) and they suggest that the density of sympathetic nerves innervating WAT is one of the key features for the browning effect to take place [81]. However, very little is known about SNS innervation in WAT browning and beige adipocytes, underlining it as one important future research line [21].

### β-3 adrenergic receptor agonists

In early rodent models of Ghorbani et al. (1997), chronic treatment with beta3-adrenoceptor agonist CL 316243 was shown to lead to occurrence of brown adipocytes within traditional WAT depots including mesenteric, inguinal, epididymal, and retroperitoneal fat depots [24]. Cousin et al. (1992) reported increase in UCP1 expression in periovarian WAT depots in rodents as a result for treatment with BRL 26830A [13].

### Short-chain fatty acids

These are end products of microbial fermentation of carbohydrates and starch by colon and have been linked to changes in body weight, appetite, and adiposity. Namely, acetate, butyrate, and propionate have been the compounds of interest while demonstrating to have protective effect against diet-induced obesity and insulin resistance in mice models [41]. Acetate has been demonstrated with WAT browning capacity in mice model [65].

### Dietary factors and organic compounds

Several plant-based compounds and dietary factors have been demonstrated to have browning effects based on studies done on mice or in vitro [85]. Capsaicin (and related capsinoids) has been demonstrated to initiate WAT browning in mice by several different mechanisms, including modulation of expression of PPAR*γ* 1 co-activator *α*, facilitation of PPAR*γ*–Prdm16 interaction, and promotion of sirtuin-1 (SirT1) expression [4]. Plant-produced resveratrol has been demonstrated to induce Ucp1 and Pgc1*α* gene expression, having similar effects to butein in a mice model [2]. Plant-derived berberine (BBR) has been shown to induce development of brown-like adipocytes in inguinal, but not epididymal adipose depots in mice through PGC-1α signaling [94]. Fish oil intake has been shown to lead to upregulation of UCP1 and the β3 adrenergic receptor in both interscapular BAT and inguinal WAT of mice [31]. In the study of Chen et al. (2017), decaffeinated green tea extract supplemented for mice induced expression of variety of browning-related biomarkers, including PPARγ, PRDM-16, BMP-7, FGF-21, UCP-1, CPT-1 CIDEA, and PGC-1α [10].

In cell culture models, cinnamon extract has been demonstrated to increase expression of brown adipocyte markers, decrease those of WAT, and induce BAT phenotype in cell culture models done with 3T3-L1 adipocyte cell line. In addition, cinnamon effected UCP1 expression in adipocytes isolated from mouse subcutaneous WAT [35]. Similarly to cinnamon, several other compounds have been studied to promote browning of 3T3-L1 cells as observed by significant increases in mRNA expressions of UCP-1, PGC-1α, and PRDM16, including ginsenoside Rb1, curcumin, and quercetin [40, 43, 51].

### Nuclear receptors and ligands

Bile acid-activated nuclear receptor, farnesoid X receptor (FXR) regulates bile acid, lipid and glucose homeostasis, and several other regulatory functions in the intestinal tract. FXR activation with fexaramine antagonist has been demonstrated to enhance thermogenesis and browning of subcutaneous WAT [19]. Liver X receptors (LXRs), nuclear receptors that control lipid and glucose metabolism, have been reported to be able to regulate the browning of WAT, activation of mitochondria, and increased energy expenditure [49].

### MicroRNAs

Different microRNA types and their roles have been studied also in terms of WAT browning and adipose tissue physiology. MicroRNAs have been shown to regulate both BAT activation and subcutaneous WAT browning in cold exposure [70]. miRNA-32 inhibition in vivo has been demonstrated to reduce FGF21 expression and compromise tolerance to cold, thus having a role in cold-induced WAT browning [52]. MiRNA-455 has been studied to enhance WAT browning response to cold and norepinephrine stimulation [92]. In contrast, several miRNA types act as negative regulators of WAT browning and BAT activity [70].

### Drug agents

Chronic treatment with thiazolidinediones (TZDs) has been demonstrated to induce browning of WAT by activating PPARγ and PRDM16 [60]. Prostaglandin E_2_ (PGE2) is a PPARγ co-operator that has been demonstrated to divert preadipocyte differentiation in WAT to beige/brite mature adipocytes and exert upregulation of UCP1 [23]. Another PPARγ-signaling affecting drug, PPARγ antagonist Gleevec has been demonstrated to significantly increase brown/beige fat thermogenic and mitochondrial genes, including *Ucp-1*, *Pgc-1*α, and *cox-5b* in WAT and interscapular BAT in mice [11]. Slit 2 derived secretory product, a member of the Slit extracellular protein family, has been demonstrated to regulate beige adipocyte induction via Prdm16 and robust activation of PKA signaling. Slit2-C fragment has an active thermogenic property having an effect on energy expenditure, and glucose homeostasis in vivo [73]. Artepillin C (ArtC) has been demonstrated to induce brown-like adipocytes in mice and primary inguinal WAT-derived adipocytes in vitro through PPARγ and PRDM16 signaling [54].

Adrenomedullin 2 (AM2) has been demonstrated to induce browning of rat primary adipocytes in vitro along with the variety of metabolism ameliorating effects through PGC1α and PRDM16 signaling [93]. Beta-lapachone (BLC) has been reported with the ability to stimulate browning of WAT, increase expression of UCP1, and ameliorate metabolic parameters of mice fed a high-fat diet. The effects of BLC were partially controlled via the regulation of miR-382 targeting deionidase iodothyronine type II (Dio2), but the overall mechanism remains unclear [12].

### Inflammatory factors

In a setting of post burn injury, bone marrow originated interleukin 6 (IL-6) has been demonstrated to regulate WAT browning [1]. Interleukin 4 (IL-4) has been reported with a role in BAT activation responding to cold exposure in a macrophage-dependent manner [53]. In addition, deficiency of immediate early response gene X-1 (IEX-1), a downstream effector of Nf-Kb signaling pathway, was demonstrated to induce browning and activate thermogenic gene program in WAT through promotion of alternative activation of adipose macrophages [69].

### Hormonal factors

Many different hormones released from variety of organs also exert a crosstalk with adipose tissue physiology and related processes. Thyroid hormones are well known to be able to induce WAT browning through both peripheral and central mechanisms [87]. Triiodothyronine (T3) metabolite triiodothyracetic acid (TRIAC) has been demonstrated with the ability to regulate WAT browning process and induce ectopic expression of UCP1 in abdominal WAT depot of rats [48]. In human cell culture model, T3 treatment enhanced UCP1 expression and mitochondrial biogenesis in human adipose-derived stem cells [37]. Circulating T4 levels have been shown to correspond to UCP1 expression in WAT in humans [47]. While deionidase type II (Dio2) is a key enzyme regulating thyroid hormone signaling and plays a crucial role in thyroid-hormone-related activation of BAT, it is also suggested to be one of the major players in thyroid hormone induced WAT browning [86]. Parathyroid hormone (PTHr) and tumor-derived parathyroid hormone-related protein (PTHrP) has been demonstrated to lead WAT browning in cancer-cachexia models studied in mice [32, 75]. Glucagon-like peptide 1 (GLP1) has been demonstrated to stimulate BAT thermogenesis and adipocyte browning in mice [5, 44]. Leptin together with insulin has been reported to drive WAT browning and weight loss through action on hypothalamic neurons [16]. Melatonin induces browning of WAT in inguinal WAT of rats as observed by increased expression of PGC-1α and UCP-1 [28]. Natriuretic peptides (NPs), endocrine hormones released from the heart, are demonstrated to possess WAT browning capacity and effect on BAT metabolism through several different pathways [6, 42].

### Genetic factors

A set of genes have been associated with WAT browning including PTEN, Cox2, Foxc2, folliculin, and G_q_. Expression of G_q_, a type of G protein-coupled receptor in human WAT, has been demonstrated to inversely correlate with UCP1 expression [33]. Modulation of TGF-β/Smad3 signaling is proposed to activate a brown adipocyte like phenotype in rodent WAT [90].

### Batokines

Batokines, such as fibroblast growth factor 21 (FGF21), interleukin 6 (IL-6), and prostaglandin D2 synthase (PTGDS), have been demonstrated to regulate glycaemia and metabolism in animal models by further enhancing glucose and lipid clearance [59, 79]. Fisher et al. demonstrated that in addition to having ameliorating effect on classical BAT and metabolism, FGF21 expression is also necessary for the cold-induced recruitment of beige adipocytes in WAT. Browning is connected with induction of PGC-1α protein levels having only little effect on mRNA expression. Since circulating levels of FGF21 are not elevated upon cold exposure, it has been suggested that FGF21 exerts its action on adipose tissue browning via para- and autocrine mechanisms [20]. However, Keipert and Jastroch demonstrated that FGF21 secreted from muscle acts as an endocrine hormone, targeting other tissues than the tissue of origin. In WAT, FGF21 secreted from skeletal muscle manifests effects that lead to the increased browning of epididymal and subcutaneous fat depots in mice [30]. Apelin, a newly identified adipocyte-derived hormone that is secreted by both brown and white adipocytes, stimulates adipose browning [74]. In their studies, Than et al. demonstrated that apelin increases the activity of BAT, induces browning of WAT, and stimulates metabolic activity both in vitro and in vivo [74].

### Exercise

Capability of exercise to induce browning has been a controversial issue. Older studies in humans and rodents report negative results for BAT activation and browning in response to exercise [68, 72, 82]. However, exercise has also been demonstrated to increase expression of PGC-1α [80, 89] and a recently identified exercise adipomyokine irisin has been shown to induce browning in human and rodents [7, 39]. Irisin is cleaved from fibronectin type III domain-containing protein 5 (FNDC5) expressed on the surface of skeletal muscle and WAT, and secreted into circulation. In their studies, Boström et al. demonstrated the browning capability of irisin in in vitro experiments in mouse subcutaneous WAT as well as described the elevated level of plasma irisin in humans in response to exercise [7].

### PPAR ligands and signaling

In mice, treatment with PPAR agonists has been demonstrated to increase UCP1 expression in different WAT depots, particularly in the inguinal depot in vivo [22, 36]. In addition, Petrovic et al. demonstrated browning of epididymal WAT depot in mice in response to prolonged treatment with PPARγ agonist rosiglitazone [60]. Study by Ohno et al. (2012) suggested that browning effect of PPAR ligands is centered to stabilization and expression of PRDM16, the main factor controlling classical BAT development [55]. On the other hand, other suggested regulators of PPARγ signaling include α/β-hydrolase domain 6 (ABHD6) that has been demonstrated to negatively regulate adipose tissue browning [95]. In cell culture models of human and mice white adipocytes, a selective PPARα-agonist WY14643 was shown to stimulate mitochondrial biogenesis and UCP1 expression along with the PPAR co-activator PGC-1α [3, 76, 78]. Since PPARγ signaling is one of the main switches in adipocyte development, its involvement in pathways related to WAT browning is highly likely.

### BMPs

Bone morphogenetic protein 7 (BMP7) was demonstrated to be able to induce BAT selective genes and induce *UPC1* mRNA expression in brown preadipocytes [77]. It also drives the browning of mesenchymal progenitor cells by inducing the expression of PRDM16. This leads to the full activation of BAT gene expression pattern, suggesting that BMP7 is one of the factors determining the cell fate between brown and white [78]. Both BMP7 and BMP4 were demonstrated to induce white-to-brown shifting in primary human adipose stem cells [18]. Bone morphogenetic proteins (BMPs) have been suggested to facilitate adipogenetic differentiation by induction of PRDM16 and PGC-1α [77] and by negatively regulating skeletal myogenesis [71]. The Wingless pathway (Wnt) is crucial in embryonic muscle formation. Activation of this pathway represses brown and white adipogenesis by suppressing the induction of PPARγ and C/EBSα in the precursor cells [64].

### Metabolites

Lactate and β-hydroxybutyrate have been demonstrated to increase *UCP1* gene expression in human and murine WAT by intracellular redox modifications [8]. Beta-aminoisobutyric acid (BAIBA) has been demonstrated to increase expression of BAT marker genes in WAT of mice both in vitro and in vivo through PPARα-mediated mechanism. In humans, plasma levels of BAIBA are increased with exercise and compound is able to induce BAT-like phenotype in human pluripotent stem cells [63]. Eicosapentaenoic acid has been demonstrated with potential to activate and increase marker gene expression in BAT depots of mice, but no effect on WAT depots has been described [58]. Retinoic acid, a bioactive metabolite of vitamin A, has been reported to induce WAT browning via activating vascular endothelial growth factor (VEGF) signaling in vitro. In co-operation with VEGFA, retinoic acid also was shown to activate p38MAPK signaling pathway and to bind to *Prdm16* promoter enhancing its transcription [83].

## Concluding remarks

Browning of white adipose tissue has been—and remains to be—an active field of research due to its demonstrated connection and potential to contribute to treatment and prevention of diabetes type 2 and other prevalent metabolic disorders. From the continuously expanding list of novel browning agents, naturally occurring compounds such as metabolites, dietary, hormonal, and stimulated physiological factors hold a special interest due to lack of side effects and non-invasive treatment possibilities of obesity and metabolic disorders.
